# Urbanization and Income Inequality in Post-Reform China: A Causal Analysis Based on Time Series Data

**DOI:** 10.1371/journal.pone.0158826

**Published:** 2016-07-19

**Authors:** Guo Chen, Amy K. Glasmeier, Min Zhang, Yang Shao

**Affiliations:** 1 Department of Geography, Environment, and Spatial Sciences & Global Urban Studies Program, Michigan State University, East Lansing, Michigan, United States of America; 2 Department of Urban Studies and Planning, MIT, Cambridge, Massachusetts, United States of America; 3 School of Architecture and Urban Planning, Nanjing University, Nanjing, Jiangsu, China; 4 Geography Department, College of Natural Resources and Environment, Virginia Polytechnic Institute and State University, Blacksburg, VA, United States of America; Feng Chia University, TAIWAN

## Abstract

This paper investigates the potential causal relationship(s) between China’s urbanization and income inequality since the start of the economic reform. Based on the economic theory of urbanization and income distribution, we analyze the annual time series of China’s urbanization rate and Gini index from 1978 to 2014. The results show that urbanization has an immediate alleviating effect on income inequality, as indicated by the negative relationship between the two time series at the same year (lag = 0). However, urbanization also seems to have a lagged aggravating effect on income inequality, as indicated by positive relationship between urbanization and the Gini index series at lag 1. Although the link between urbanization and income inequality is not surprising, the lagged aggravating effect of urbanization on the Gini index challenges the popular belief that urbanization in post-reform China generally helps reduce income inequality. At deeper levels, our results suggest an urgent need to focus on the social dimension of urbanization as China transitions to the next stage of modernization. Comprehensive social reforms must be prioritized to avoid a long-term economic dichotomy and permanent social segregation.

## Introduction

Rapid urbanization and rising inequality are two main characteristics of China’s development over the past three plus decades. Since the start of the open and reform policy in the late 1970s, the number of urban inhabitants in China has ballooned from 172 million (or 18% of the total population) in 1978 to 749 million (or 55% of the total population) in 2014 [1]. The massive urban transition has not only fueled China’s burgeoning economy but also helped modernize Chinese society as a whole. In parallel to the country’s rapid urbanization is widening income inequality. Although the post-reform economic growth has been praised for lifting millions of (rural) Chinese out of absolute poverty [2–4], it has also contributed to income stratification due to uneven developmental policies [5–8] and the persistent disparity in the distribution system [8]. According to official statistics, China’s income Gini index increased from 0.3 in 1978 to around 0.5 in 2014 [1], and a recent survey estimates that the national wealth Gini index could be as high as 0.73 [9].

Whether rising urbanization and inequality are causally related becomes a natural question given their co-occurrence. Classical developmental theories, e.g., those popularized by W. A. Lewis and by S. Kuznets, consider urbanization a key step in reshaping emerging economies dichotomized by a subsistence rural sector and an industrializing urban sector. The rural-to-urban population shift is an aspect of the processes behind the famous inverse-U-shaped curve hypothesized by Kuznets, who argues that as more people move from the lower-income rural sector to the higher-income urban sector, the overall income inequality will first increase and then decrease [10]. It is believed that China has already passed the turning point and, therefore, urbanization should help alleviate income inequality [11]. This assessment seems to be consistent with the fact that the overall income inequality in China can be attributed in large part to the rural–urban income gap [12], and urbanization can potentially reduce the impact of the gap (if not narrow it) by absorbing more people into the higher-income urban sector [13].

However, to date this theoretical assessment has only been empirically tested in a limited way. After all, co-occurrence (and correlation) does not equal causation and no previous studies have empirically established or refuted the causal relationship between urbanization and income inequality in China. In this paper, we address this blank by analyzing the annual time-series of China’s urbanization rate and Gini indexes since 1978. We adopt the inference framework of predictive causality widely used in econometrics and employ autoregressive methods to model the potential causal relationships. The purpose is to establish a parsimonious yet predictively verifiable baseline causal link between urbanization and income inequality, which offers important insights for China’s urban studies and policy-making. The implications of our results are then discussed in the context of post-reform China.

## Theoretical Framework

### Causal Effect of Urbanization on Income Inequality

**Fig 1:**
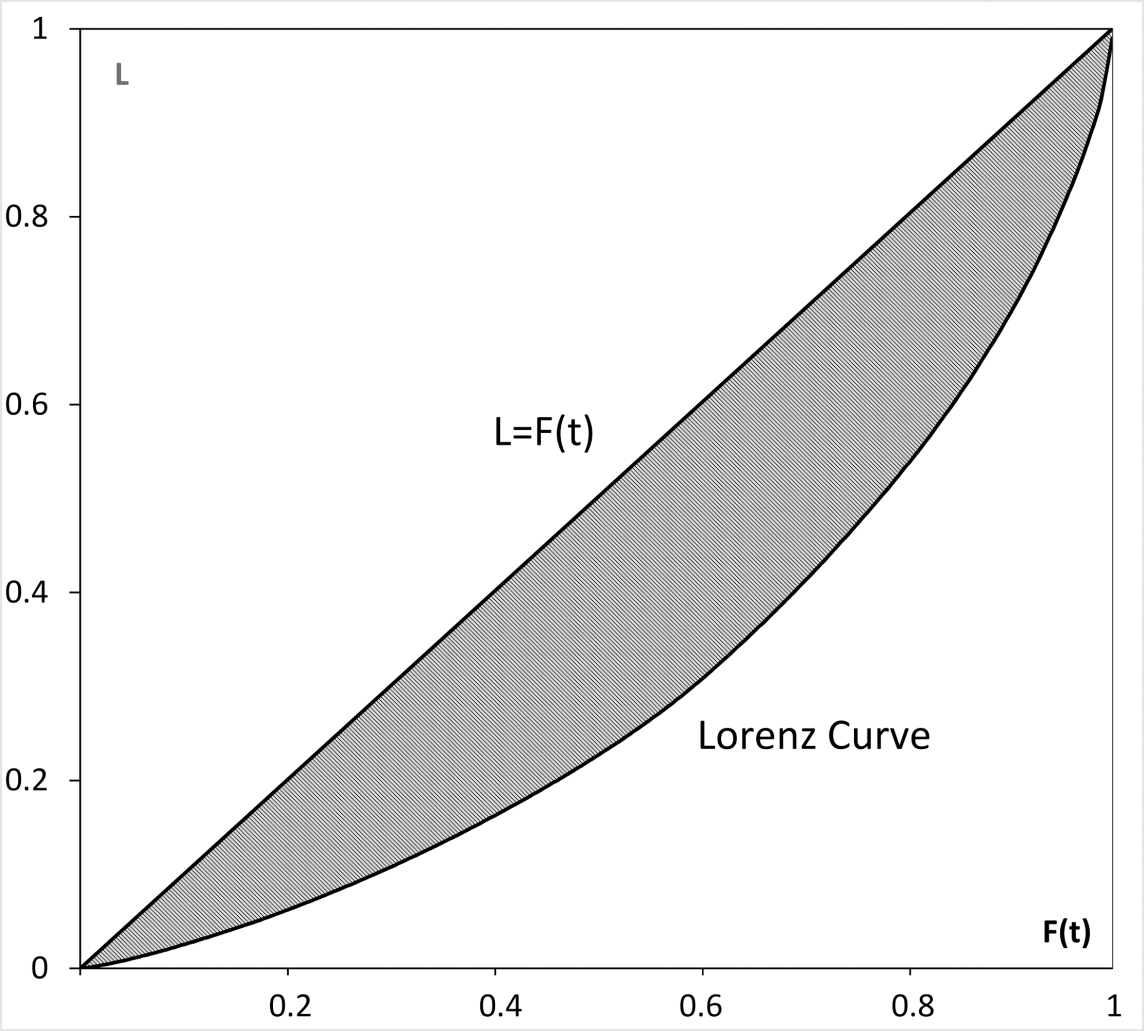
Lorenz curve and the Gini index. The value of the Gini index equals the area of the shaded gray region.

We measure income inequality using the popular Gini index, which is mathematically based on the Lorenz curve [14]. Suppose the cumulative income-to-population distribution function of a country is *F(t)*, where *t* is a random variable of personal (or household) income and *F(t)* is the share of population (or households) earning less than or equal to *t*. The Lorenz curve is depicted in Fig 1, where the vertical axis *L* denotes the cumulative income of the population *F(t)*. The Gini index is the area between the Lorenz curve and the straight line *L = F(t)* (the shaded gray area in Fig 1) multiplied by 2.

First, we follow steps illustrated in previous studies [15–17] to derive the function from the income variable *t* to the Gini index *G*. Based on the definition of the Lorenz curve, we have

$$L\left(F(x)\right)\;=\;\frac{1}{V}\int_{t=-\infty }^{x}tf(t)dt\;=\frac{1}{V}\int_{t=-\infty }^{x}tdF\left(t\right)$$ (1)

and

$$dL\;=\;\frac{1}{V}tdF$$ (2)

where *V* denotes the average income and *f(t)* denotes the income probability density function. Let *B* denote the area between the Lorenz curve and the horizontal axis such that we have

$$B\;=\;\int_{F=0}^{1}L\;dF$$ (3)

By integrating by parts and replacing the integral bounds, we have

$$B\;=\;{\left[LF\right]}_{F=0}^{1}-\;\int_{L=0}^{1}FdL\;=\;1-\;\int_{L=0}^{1}FdL$$ (4)

After substituting (2) into (4), changing the integral bounds to those of *t*, and integrating by parts, we have

$$B\;=\;1-\;\frac{1}{V}\int_{t=-\infty }^{+\infty }tF\left(t\right)dF(t)\;=\;1-\;\frac{1}{2V}\left\{{\left[tF^{2}(t)\right]}_{-\infty }^{+\infty }-\int_{-\infty }^{+\infty }F^{2}(t)\;dt\right\}$$ (5)

To further simplify (5), it is reasonable to assume that in reality the income variable *t* is within the range [*0*, *T*], where *T* denotes the highest income. Then, we can rewrite (5) as

$$B\;=\;1-\;\frac{1}{2V}\left\{{\left[tF^{2}(t)\right]}_{0}^{T}-\int_{0}^{T}F^{2}\left(t\right)\;dt\right\}$$ (6)

Based on the definition of *F(t)*, we have *F(0) = N*_*0*_*/N*, where *N* denotes the total population size and *N*_*0*_ is the number of people (households) with zero income. As *N*_*0*_ is usually small relative to *N*, we can further assume that *F(0)* → 0 when *N* is large. Similarly, we can assume that *F(T)* approximates 1 when *N* is large. Then, (6) becomes

$$B\;=\;1-\;\frac{1}{2V}\left\{T-\int_{0}^{T}F^{2}(t)\;dt\right\}$$ (7)

Under the same assumptions, we have

$$V\;=\;\int_{t=0}^{T}tdF\left(t\right)\;=\;T-\int_{0}^{T}F\left(t\right)dt$$ (8)

Let *G* denote the Gini index and it is straightforward that

$$G\;=\;1-2B\;=\;\frac{\int_{0}^{T}F\left(t\right)dt-\int_{0}^{T}F^{2}\left(t\right)dt}{T-\int_{0}^{T}F\left(t\right)dt}$$ (9)

Next, we derive the relation between urbanization and the Gini index. Suppose the cumulative population-to-income distribution functions in the urban and rural sectors can be represented by *F*_*u*_*(t)* and *F*_*r*_*(t)*, respectively. Let *x* ∈ [*0*,*1*] denote the urbanization rate (i.e., the percentage of urban population), and let *y = 1- x* denote the percentage of rural population. We have

$$F\left(t\right)\;=\;{xF}_{u}\left(t\right)+yF_{r}\left(t\right)$$ (10)

Let *U* denote the average income of the urban population, *R* denote the average income of the rural population, *G*_*u*_ denote the urban Gini index, and *G*_*r*_ denote the rural Gini index. Based on (8) and (9), we have

$$U\;=\;T-\int_{0}^{T}F_{u}\left(t\right)dt$$ (11)

$$R\;=\;T-\int_{0}^{T}F_{r}\left(t\right)dt$$ (12)

$$G_{u}\;=\frac{\int_{0}^{T}F_{u}(t)dt-\int_{0}^{T}F_{u}^{2}(t)dt}{U}$$ (13)

$$G_{r}\;=\frac{\int_{0}^{T}F_{r}(t)dt-\int_{0}^{T}F_{r}^{2}(t)dt}{R}$$ (14)

Moreover, we define

$$D\;=\;\int_{0}^{T}{\left\{F_{u}\left(t\right)-F_{r}\left(t\right)\right\}}^{2}dt$$ (15)

By substituting (10)–(15) into (9) and letting *G' = G*_*u*_*—G*_*r*_ denote the difference between the urban and rural Gini indexes, we have

$$G\;=\;G_{r}+\;\frac{UG\prime x+Dxy}{Ux+Ry}\;=\;G_{r}+\;\frac{-Dx^{2}+\left(UG^{\prime }+D\right)x}{(U-R)x+R}$$ (16)

To study the monotonicity of (16), we consider the first derivative of *G*:

$$\frac{dG}{dx}=\;\frac{-(U-R)Dx^{2}-2RDx+R(UG^{\prime }+D)}{{\left\{(U-R)x+R\right\}}^{2}}$$ (17)

As {(*U—R*) *x + R*}^*2*^ > 0, setting (17) to 0 requires the nominator to be 0. By solving the quadratic equation

$$-(U-R)Dx^{2}-2RDx+R(UG^{\prime }+D)\;=\;0$$ (18)

we have

$$x\;=\;\frac{\pm \sqrt{UR\left\{1+\frac{G^{\prime }}{D}(U-R)\right\}}-R}{U-R}$$ (19)

Note that by definition *0 ≤ x ≤ 1* and we assume *U > R*. Therefore, $\frac{-\sqrt{UR\left\{1+\frac{G^{\prime }}{D}(U-R)\right\}}-R}{U-R}<0$ and the turning point of (16) should be at

$$X\;=\;\frac{\sqrt{UR\left\{1+\frac{G^{\prime }}{D}(U-R)\right\}}-R}{U-R}$$ (20)

As the opening of the quadratic curve is downward and *X* is the larger root of (18), we can see that when *0 ≤ x < X*, *dG/dx > 0* and the Gini index increases with urbanization. Similarly, when *X < x ≤ 1*, *dG/dx* < 0 and the Gini index decreases with urbanization. This is consistent with the inverse-U-shaped curve depicted by Kuznets. It is noteworthy that *X* is not always on [0, 1]. If *G' > D/R*, i.e., if the urban Gini index exceeds the rural Gini index plus *D/R*, then *X >* 1 and urbanization will always cause *G* to increase. On the other hand, if *G' < -D/U*, i.e., if the urban Gini index is smaller than the rural Gini index minus *D/U*, then *X <* 0 and urbanization will always cause *G* to decrease.

### Predictive Causal Model

The advantage of (20) is that it only depends on the rural and urban income distributions, as *U*, *R*, *G*_*u*_, and *G*_*r*_ are all functions of *F*_*u*_*(t)* and *F*_*r*_*(t)*. A main assumption is that both *F*_*u*_*(t)* and *F*_*r*_*(t)* remain the same after the population shift. In reality, however, this condition is unlikely to hold. Newly arriving rural migrants are concentrated in the lower-income strata and/or informal sectors of the urban economy, which tends to downwardly skew *F*_*u*_*(t)*. It takes time for *F*_*u*_*(t)* to recover and the extent to which *F*_*u*_*(t)* can recover depends on a range of factors, including the structure of the urban economy, the extent of social mobility, and access to opportunities for the migrant groups. Moreover, some of the impact of urbanization could be long-lasting and/or lag over time. For example, adding needed labor to a fast-growing urban sector can accelerate economic expansion, whereas barriers to opportunities can trap rural migrants in persistent poverty [18, 19]. In both situations, income distribution will shift and income inequality may change over time.

For these reasons, in reality the impact of urbanization on income inequality is a complex process that may span multiple years. Under the traditional causal inference framework based on *ceteris paribus* (i.e., all other factors equal), addressing such dynamics requires highly complex structural models (or systems of models) with a large number of parameters. In most situations, however, such models are difficult to correctly specify and/or efficiently estimate due to limitations in data availability, measurement quality, and model statistical power. Moreover, such models are highly sensitive to misspecification. It is not uncommon that a dynamic structural model with many parameters is not immediately identifiable and additional assumptions are needed to restrict the parameter space. As argued by C. Sims, these requirements not only obfuscate the logic of causal inference but they can also produce highly misleading results, as the numerous assumptions imposed by the structural model are not necessarily warranted in real-world analysis [20].

The concept of predictive causality, largely pioneered by Clive Granger [21], offers an alternative approach to causal inference in dynamic domains. In this approach, causality between two processes is defined by whether observations of one can help predict the other. Compared with *ceteris paribus* this is a weaker causality framework, as it merely describes the temporal correlation between two time-series without controlling for other factors. In other words, the predictive causality model focuses on prediction but may not offer the same explanatory power as a traditional dynamic structural model would. However, it provides a simple yet powerful tool in practice for domains such as macroeconomic analysis, where data are often limited and detailed structural relationships are usually too complex to correctly specify and/or identify. In this study, we hypothesize that predictive causal relationships exist between urbanization and income inequality. We first use vector autoregression (VAR) to explore such relationships. Since we have a relatively small number of time points, the results of the VAR model are not intended to provide inferential evidence; instead they are interpreted exploratorily to guide the specification of a more parsimonious autoregressive model. The final inference is based on a simple autoregressive model, which requires fewer parameters and thus can be more convincingly supported by the short time series.

From the perspective of traditional structural regression, the omission of variables could be a serious problem for our analyses. For example, one may argue that any relationships between urbanization and Gini index are spurious because the omission of economic development, which affects both urbanization and income inequality. This is indeed an issue if we take the model at face value in policy-making and attempt to regulate inequality by boosting or controlling urbanization without checking the conditions of other relevant factors such as economic growth [20]. However, it is less a concern if we stick with the logic of predictive causality and treat the results only as evidence of two temporally correlated processes observed in the study time frame. In other words, our goal is to reveal the temporal relationships between urbanization and income inequality as suggested by the time series, which should not be interpreted explanatorily but could offer an informative baseline to elicit more detailed explanatory studies.

## Data and Methods

The official counts of urban population in China have historically been subject to inconsistencies in regard to both the definition of urban resident and the protocols used to collect data [22]. Since 2000, Chinese authorities have adopted a more sensible and internationally comparable definition of urban population, which counts all permanent residents (i.e., those who have lived locally for at least 6 months) in urban areas defined by socioeconomic activities rather than administrative boundaries [22]. The annual urbanization data before 2000 have since been revised using the new definition and based on the results of the population censuses. In this study, we use the latest revised urbanization data series, which is now included as the official data in the NBSC’s most recent publications.

The Chinese authorities did not consistently publish official estimates of the national Gini index before 2003, although the NBSC has a history of reporting the average disposable (or net) incomes of different urban and rural income strata estimated based on household surveys. In addition to the NBSC’s surveys, income data are also available from other national surveys, e.g., those administered under the China Household Income Project (CHIP) [23] and the Chinese Family Panel Studies (CFPS) [24]. Unfortunately, only the NBSC surveys provide a relatively complete data series since 1978. In the related literature, a common method used to obtain the annual Gini index series is to fit the urban and rural income distributions based on the NBSC data. In the present study, the Gini indexes from 1978 to 2006 were taken directly from Chen (2010), who used the lognormal functions to fit the grouped income data from the NBSC’s urban and rural surveys [25]. Estimates for the years after 2006 were calculated based on lognormal functions fitted by the authors using the NBSC data. We also used Gini index estimates from other studies to cross-check our results. It turned out that although different estimation methods produced different Gini index values for individual years, the methods generated similar trend patterns for the time series.

We followed the standard procedure outlined in the related literature to conduct the time series analyses [26]. First, we explored trends in the time series and performed unit root tests to confirm the (non-)stationarity in the data. After the expected non-stationarity in all three variables was confirmed, we log-transformed the original variables to linearize their relationships and took the first differences of the log-transformed data. The VAR model was built using the first differences of the log-transformed variables. After that, orthogonalized impulse-response functions were obtained based on Cholesky decomposition. It is reasonable to assume that (1) urbanization may have immediate effects on income inequality and (2) the feedback from income inequality to urbanization may occur at a later time. Therefore, the variable ordering for Cholesky decomposition should be urbanization followed by inequality.

Based on the results of the VAR model, Granger causality tests were conducted as F-tests (or equivalence) on the coefficients of relevant autoregressors. After exploring the plots of impulse response functions produced by the VAR model, we built an autoregressive model with a minimal number of parameters–which only includes variables at time lags that were found to be relevant by the VAR model. The ACF and PACF plots were used to determine the necessity of the autoregressive (AR) and moving average (MA) terms. The final model was estimated using the Cochrane-Orcutt method. All the data treatments were performed in the software packages EViews [27], R [28] and gretl [29].

## Results

**Fig 2:**
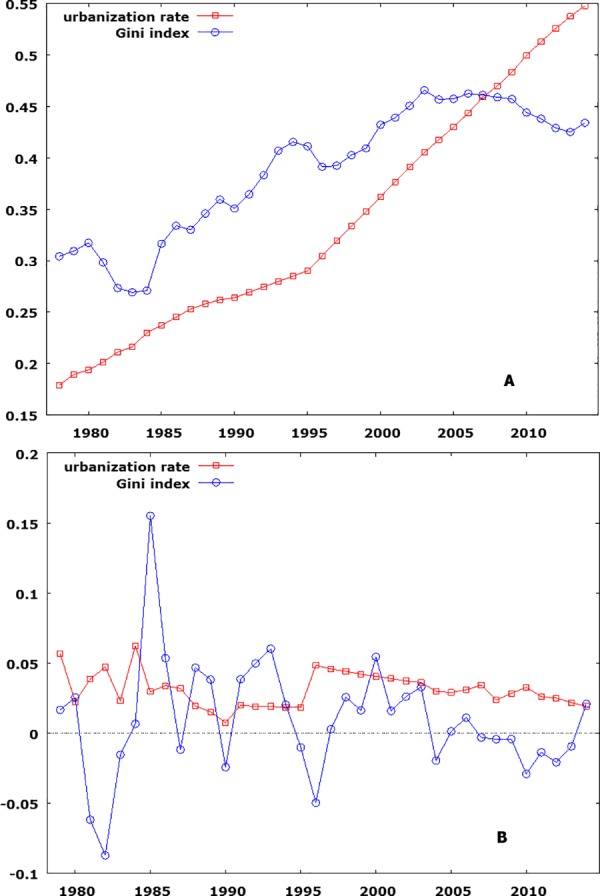
Urbanization rate and Gini index in China from 1978 to 2014. Fig 2A shows the time series in levels. Fig 2B shows the first differences of log-transformed data.

The time series of China’s urbanization rate and Gini indexes from 1978 to 2014 are plotted in Fig 2. Both processes appear to be non-stationary and the first differences of their log-transformed values appear to be stationary. These impressions are confirmed by unit root tests (Table 1). The first lull of the Gini index series in the early 1980s can be attributed to the rural decollectivization during the early stage of the reform [8, 30, 31]. But as the process of urban reform picked up pace, the Gini index’s upward trend resumed. Meanwhile, it is likely that the decrease in income inequality in the mid-1990s is related to the wage reform [32], which implemented a more merit-based reward structure and increased the wage levels in most work units [33]. However, as state-owned enterprises (SOEs) started to face drastic reform measures taken by the government that pushed many of SOE employees out of work [34–36], income inequality climbed again in the latter half of the 1990s.

**Table 1:** Results of unit tests.

	**Urbanization rate**	**Gini index**
ADF	> 0.1	> 0.1
ADF-GLS	> 0.1	> 0.1
KPSS	< 0.01	< 0.01
ADF	< 0.01	< 0.01
ADF-GLS	< 0.01	< 0.01
KPSS	> 0.1	> 0.1

For ADF and ADF-GLS tests, the null hypothesis is that the series has a unit root. For KPSS tests, the null hypothesis is that the series is stationary. The ADF and ADF-GLS tests followed a “test-down” procedure starting with a maximal lag order of 8, whereas the KPSS tests used a lag order of 3.

The Gini index’s dip after 2006, unfortunately, is less clearly understood. On the one hand, China’s national income inequality may have been alleviated by recent economic and policy changes, including the shift of manufacturing jobs from the coastal areas to the inland provinces [37], the abolition of agricultural taxes [38], and the continued development of a social safety net together with income redistribution [39–42]. After all, one cannot deny that given China’s rapid economic development, there is a possibility that the downhill part of the Kuznets curve may have been reached and that income inequality has started to decline. On the other hand, researchers remain highly suspicious of the official data, as almost all non-governmental data sources reported higher estimates of the Gini index for the same period of time than the official data sources suggest. The same kind of decline, for example, is not seen in estimates based on CFPS data [12]. To ensure the consistency of our analysis, we proceeded with the estimates based on NBSC, but used sub-samples and alternative estimates of the Gini index to cross-validate the results.

## Results of VAR Estimation

It is noteworthy that trend changes around the mid-1990s can also be observed in the urbanization rate series, which appears to climb steeply after 1995 (Fig 2A). This could be the cumulative effects of relevant transformations in China’s economic system, which started following a more city-oriented strategy to accelerate economic growth in the 1990s [8]. The full-scale housing marketization, which jump-started a fast-growing urban real-estate industry, also occurred in the second half of the 1990s [43]. Consequently, we created a dummy exogenous variable, which was assigned as 1 for the years after 1995 and 0 otherwise. A second dummy variable was created for year 1989 (i.e., 0 for all but year 1989), as observations in this year turned out to be outliers. Eventually, a VAR(2) model was selected based on information criteria, and its estimates are summarized in Table 2. Despite the relative short time series, the VAR model passed all tests for model adequacy. Consequently, the VAR model provides good exploratory insights over the data.

**Table 2:** Summary of VAR estimates.

	**Urbanization rate**	**Gini index**	
For individual equation			
R-squared	0.912437	0.458810	
Adjusted R-squared	0.892978	0.338546	
Sum of squared residuals	0.003164	0.033568	
F-statistic (7, 27)	40.19268	3.270014	
P-value	< 0.001***	0.012**	
Mean dependent	0.030539	0.009202	
S.E. equation	0.010825	0.035260	
S.D. dependent	0.011581	0.042336	
F-tests of zero restrictions (Granger causality tests)			
All lags of urbanization rate	< 0.001***	0.007***	
All lags of Gini index	0.6261	0.006***	
For the system as a whole			
OLS estimator	T = 34 (1981–2014), Log-likelihood = 180.19023		

Significance codes:

* < 0.1

** < 0.05

*** < 0.01

Model estimator: OLS with T = 34 (1981–2014)

**Fig 3:**
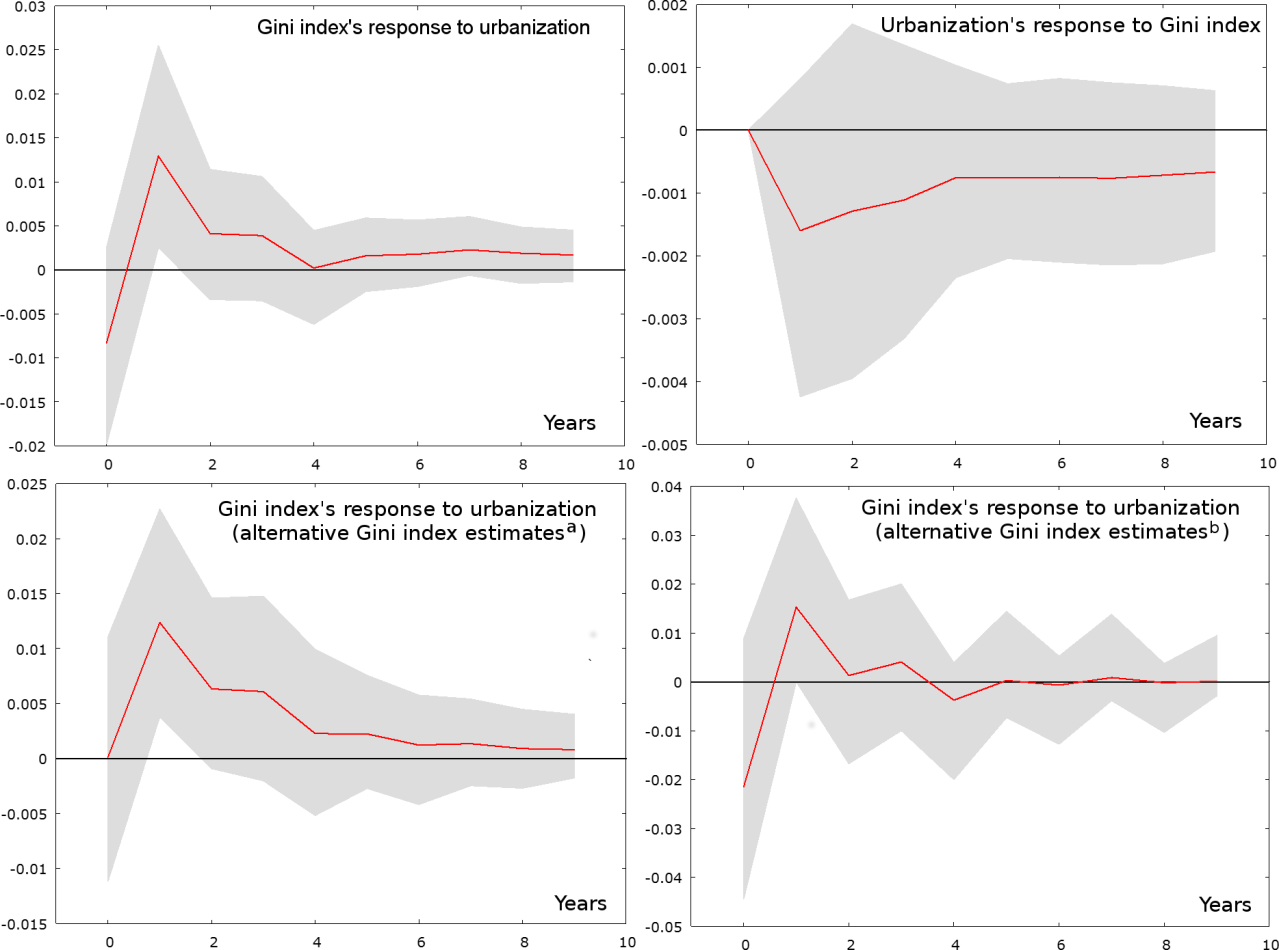
Impulse response functions with 90% intervals. ^a^ Using Gini index estimates by Cheng (2010) (Years: 1981–2004). ^b^ Using Gini index estimates by Wu and Perloff (2006) (Years: 1985–2001).

Fig 3 depicts the impacts of an unexpected unit (one standard deviation) shock in one variable on the other over time. For the Gini index, the immediate response to urbanization is negative, suggesting that the urbanization shock leads to reduced income inequality in year 0. However, a significant positive hike occurs in year 1, which means that income inequality’s response tends to increase significantly one year after the initial urbanization shock. On the other hand, urbanization’s response to the Gini index shock is not significantly different from zero. In other words, the impulse response functions suggest a one-way causal relationship from urbanization to the Gini index, which is consistent with the results of the Granger causality tests in Table 2. To show the robustness of the Gini index’s response, Fig 3 also presents impulse response functions obtained using alternative Gini index estimates [44, 45] under the same model specification. These data series contain even fewer time points than our data, and the responses they produce barely cross the 90% confidence intervals. But it is important to note that all these impulse response functions exhibit similar trend patterns. From the perspective of explorative analysis, this means that the Gini index’s response to urbanization revealed by the VAR model is unlikely to be a random artifact.

## Results of AR(1) Estimation

**Fig 4:**
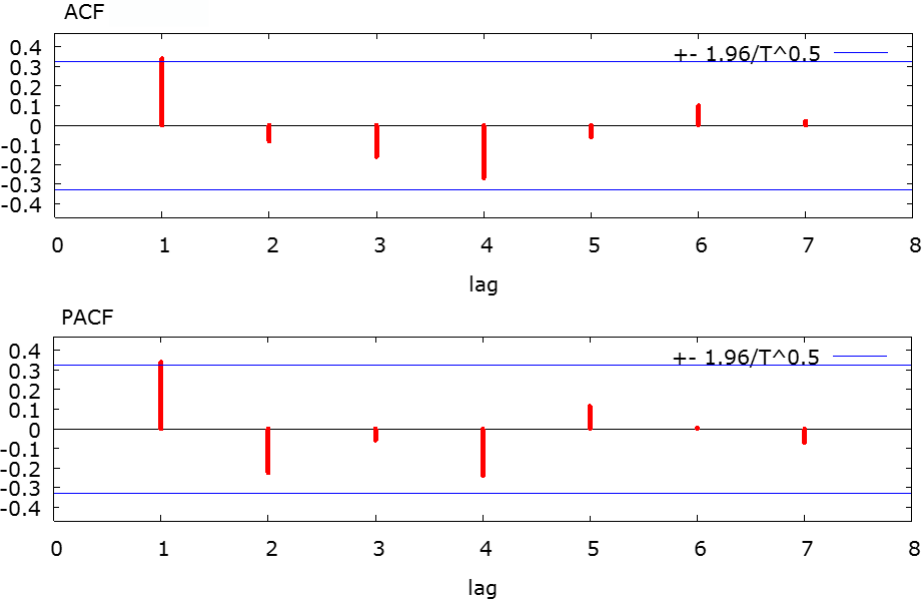
Correlogram for the first differences of the log-transformed Gini index series. ACF: autocorrelation function; PACF: partial autocorrelation function.

Based on the exploratory interpretation of the VAR results, an AR(1) model in first differences with no constant and no MA term was built for the Gini index. The correlograms for ACF and PACF for the first differences of the log-transformed Gini index series were depicted in Fig 4, which justified the inclusion of the AR(1) term and the exclusion of the MA term. According to the results of the Granger tests in Table 2, urbanization Granger-causes the Gini index but not vice versa. So we include the urbanization rates in lag 0 and lag 1 as exogenous variables. The Gini index at lag 1 was also included as a control. The results of the 3-parameter AR(1) model were reported in Table 3. All three terms were highly significant, suggesting the Gini index in any year is dependent on its value in the previous year as well as the urbanization rates in both the same year and the previous year. The signs of coefficients for the two urbanization rate variables are consistent with what the VAR model depicts, i.e., negative in year 0 and positive in year 1. Consequently, there is high statistical confidence that urbanization’s predicative causal effects on income inequality are not artifacts.

**Table 3:** Summary of AR(1) estimates.

	**Coefficient**	**Std. Error**	**p-Value**
Urbanization rate in the same year (lag 0)	−1.24793	0.586921	0.0416**
Urbanization rate in the previous year (lag 1)	1.55939	0.501417	0.0040***
Gini index in the previous year (lag 1)	0.19676	0.366520	0.5952

Significance codes:

* < 0.1

** < 0.05

*** < 0.01

Dependent variable: Gini index (first difference of log-transformed values); Model estimator: Cochrane-Orcutt with T = 34 (1981–2014); Normality test of residuals: p-value = 0.32 (null hypothesis of normal distribution)

## Discussion

The Gini index’s lagged positive response indicates that urbanization may aggravate income inequality over time—a point that should be addressed as China’s marketization continues to move forward. It is possible to identify three general directions for more detailed explanatory research from the perspectives of regional inequality, the rural–urban gap, and the persistent marginalization of rural migrants.

### Potential Aggravating Factors of Inequality

First, urbanization may have worsened regional inequality. Regional development in China is known to follow a laddered geographic pattern, where the fast-growing coastal areas and the less-developed inland provinces (or the eastern, central, and western regions in a tri-partite view) form distinct growth “clubs” [46]. It has been shown that economic development has converged within each club, which helps suppress regional inequality at the national level [47]. But the inter-club gap seems to be persistent despite the central government’s recent efforts to accelerate inland development [48]. As migrants from the inland rural areas have been an important force for both the urbanization and economic development of the coastal regions, it is reasonable to hypothesize that urbanization may have causally increased the income gap between the origin and destination of the migration due to the widening difference in economic growth. Note that this process is probably more subtle than coarse scale statistics (e.g., average GDP and/or income at the provincial level) can indicate, because it is highly dependent on the structure of local urbanization, the conditions of the local labor market, and specific migration flows.

Second, the lagged positive response in the Gini index may reflect a growing difference in productivity between the rural and urban sectors. Despite the success of the rural decollectivization in the early stage of China’s economic reform, the incentives for farming have gradually languished due in part to high taxes and fees and relatively low returns [49]. Moreover, cropland in rural China is tenured through the household responsibility system (HRS), under which land is owned collectively but controlled by individual households. Although HRS boosts household-level productivity and protects land security for peasants [50], it limits technological advancements and economies of scale due to ambiguous property rights [51], the under-developed land rental market [52], and the lack of adequate financial services for individual farmers [53]. As the urban economy continues to outpace its rural counterpart, the opportunity cost for farming rises and the most productive group of the rural population (e.g., young, healthy, and educated adults) continue to leave for the cities. Consequently, a structural shortage of human resources has emerged in some rural areas [54] and the rural sector lags further behind the booming urban sector in terms of productivity. Again, this process may be difficult to identify using aggregated data given the variation in population flows and local labor markets.

Third, urbanization’s lagged effect on income inequality could be the result of the persistent marginalization of the rural migrants. Rural migrants in China are known to face additional hurdles set up by discriminatory institutions, especially those related to the Hukou household registration system [55], which ties people’s access to services to their residential status. Due to the institutionalized measures whereby migrants are deprived of job opportunities, educational rights, and social welfare benefits, the marginalization of a rural migrant usually lasts for an extended period of time [56]. Consequently, urbanization can lead to a ballooning low-income group that further widens urban income inequality, and the lack of integration with the mainstream urban society can trap migrant groups in segregated spaces and long-term poverty [19, 57]. Although a well-recognized problem in the related literature, the persistent poverty of rural migrants is poorly documented in many official and unofficial data sources, as most general-purpose surveys tend to undersample the migrant population [58]. Future research on this subject, therefore, must draw extensively on more representative and preferably firsthand data sources.

### The Importance of Social Reform

It is noteworthy that some of the above problems have been addressed to some extent in recent years. The relocation of the manufacturing industry from the coastal areas to the inland provinces [37] has the potential to reduce urbanization’s aggravating effects on regional inequality, as many rural migrants can now find jobs in nearby cities. The abolition of the agricultural tax [38] and the continued development of the cropland rental market help boost agricultural productivity [52]. There are also advances in health care and social welfare reforms, which have greatly expanded the population protected by the emerging social safety net [39, 42, 59]. A main challenge at this moment, however, is probably the Hukou reform. Although restrictions on employment and residential mobility have gradually withered as economic marketization has deepened, the role of Hukou as a social divider continues to prevail. The ongoing effort to extend social and welfare services to non-local Hukou holders has been beleaguered by the lack of an affordable and accountable solution. Currently, local governments at the receiving end of urbanization are asked to carry out the reform and absorb its enormous cost, which has created significant budgetary pressure on the cities. As the recent slowdown in China’s real estate sector has worsened widespread local debt situations [60], real progress in regard to the Hukou reform is at a standstill in most parts of China.

From a broader policy perspective, the problems of assimilation and integration caused by long-standing discriminatory institutions have resulted in a permanent dichotomy in the social and economic system, which have already obstructed China’s progress toward the second stage of the Lewis model. For example, many rural migrants have to leave dependents, including women, children, and aging parents, in the countryside because of the cost and restrictions imposed by the Hukou system on housing, education, and retirement arrangements. The resulting separation of families, which is estimated as affecting more than 60 million children, is creating profound social, psychological, and human development problems that may haunt Chinese society for generations to come [61]. Due to the high stakes nature of this issue, it is important for both central and local governments to design and effect a more comprehensive, cross-region, and cross-administrative-level reform strategy, as problems such as those related to Hukou cannot be solved by the central or local government alone.

In summary, a broad message from this study is the importance of comprehensive social reforms. Currently, the discussion on China’s urbanization is preoccupied by two dominant theses: the pro-growth thesis that considers urbanization an economic engine, and the pro-sustainability thesis fueled by reactions to problems created or exposed by urbanization. In our opinion, both theses, in their current forms, lack a social dimension, and as a result, the ongoing policy debate is often out of touch with the people directly affected by the urban transition. Pro-growth urbanization policies embraced by GDP-chasing local governments, for example, have led to land-oriented rather than people-centered urban expansion, creating problems ranging from urban sprawl to environmental degradation [62]. On the other hand, the pro-sustainability argument often mistakenly places the blame for environmental woes on the growing urban population, ignoring the fact that expanding land development and increasing consumption among the existing urban residents are probably more culpable than any effect wrought by newcomers. Addressing the income-inequality problem helps reintroduce social justice into the discussion, which constitutes an important first step toward a more socially balanced urbanization policy.

## Conclusion

In summary, this study shows that the rising income inequality in post-reform China can be causally associated with urbanization. Time series analyses indicate that urbanization has a non-linear and time lagged effect on income inequality, as it causes the Gini index to initially decrease but eventually increase. The lagged aggravating effect of urbanization on income inequality points to an important direction for future research, which can be approached from the perspectives of regional inequality, the rural–urban income gap, and the persistent marginalization of rural migrants. These results highlight the lack of a social dimension in China’s urbanization policy, particularly the stagnation of the Hukou reform. Due to the profound impact this problem may have on China’s economic and social system, more comprehensive social reforms must be prioritized in order to redress the widening income inequality during urbanization and address the discriminatory institutions behind it.

## Supporting Information

S1 File Urbanization rates and Gini index estimates in China from 1978 to 2014. (CSV)
